# Proposed Clinical Practice Guidance for Large-Volume Abdominal and Pleural Paracentesis with Emphasis on Coagulopathy Management

**DOI:** 10.3390/jcm14238287

**Published:** 2025-11-21

**Authors:** Carmi Bartal, Emanuel Sikuler, Philip Tsenter, Vitali Perski, Valery Dvorkin, Roman Pairous, Doron Schwartz

**Affiliations:** 1 Internal Medicine Daycare Unit, The Soroka Medical Center and Ben-Gurion University of the Negev, Beer-Sheva 8400101, Israel; vitalype@clalit.org.il (V.P.); valery770@gmail.com (V.D.); tzilapa@gmail.com (R.P.); 2 Gastroenterology and Hepatology Institute, The Soroka Medical Center and Ben-Gurion University of the Negev, Beer-Sheva 8400101, Israel; emanuels@clalit.org.il (E.S.); doronsh@clalit.org.il (D.S.); 3 Risk Management Unit, The Soroka Medical Center and Ben-Gurion University of the Negev, Beer-Sheva 8400101, Israel; 4 Respiratory Diseases Institute, The Soroka Medical Center and Ben-Gurion University of the Negev, Beer-Sheva 8400101, Israel; philiptsenter@gmail.com

**Keywords:** large-volume paracentesis, ascites, pleural effusion, coagulation management, ultrasound guidance

## Abstract

**Background**: Large-volume paracentesis (LVP) of the peritoneal and pleural cavities is a common diagnostic and therapeutic intervention in patients with liver cirrhosis or advanced heart failure, which are both frequently associated with ascites or pleural effusion. Although generally regarded as a low-risk procedure, LVP may lead to complications such as intrapleural or intra-abdominal hemorrhage, and more commonly abdominal wall bleeding, as well as organ puncture and infection. Performing LVP in patients with coagulopathy or bleeding disorders, whether disease-related or due to anticoagulant therapy, poses a significant clinical challenge. The safety thresholds for such procedures remain inconsistent, and strategies to mitigate bleeding risk are still debated among professional societies. **Methods**: This review integrates institutional experience with a systematic synthesis of the current international literature to identify the safest and most effective approaches for performing LVP in patients with coagulopathy. The methodological framework included a comparative analysis of existing professional guidelines, as well as a critical evaluation of published evidence regarding risk stratification, pre-procedural correction strategies, and peri-procedural management. The evidence grading was assessed with the STAIR checklist. **Results**: Analysis of the evidence revealed substantial variability among professional recommendations concerning acceptable platelet and INR thresholds, as well as differing approaches to the management of patients receiving anticoagulant or antiplatelet therapy. Despite these discrepancies, the aggregated data support the conclusion that LVP can be performed safely in most patients with mild-to-moderate coagulopathy, provided that appropriate risk assessment and technical precautions are implemented. **Conclusions**: The resulting evidence-informed suggestions provide a practical framework for clinicians performing LVP in high-risk patients. By emphasizing systematic pre-procedural evaluation, individualized management of coagulopathy, and adherence to standardized procedural techniques, this work aims to promote safety, consistency, and confidence in the performance of large-volume paracentesis across diverse clinical settings.

## 1. Introduction

Ascites and pleural effusion are common manifestations of decompensated liver cirrhosis and congestive heart failure (CHF). Large-volume paracentesis (LVP) is often required for symptom relief, prevention of complications such as spontaneous bacterial peritonitis (SBP), and diagnostic evaluation. The balance between bleeding risks and thrombosis in patients with cirrhosis and CHF complicates decision-making, particularly when coagulopathy or anticoagulant therapy is required. Various professional societies, including the Gastroenterological Association and the Society of Interventional Radiology (SIR), have proposed clinical guidelines, which may create uncertainty in clinical practice. The motivation for this work arose from discrepancies among the guidelines of the professional societies regarding peritoneal and pleural effusion, especially regarding the pre-procedural management of coagulopathy. Given the inconsistencies, this study reviewed the current literature and proposed a unified set of practical suggestions designed to enhance procedural safety, reduce variability in practice, and promote standardized, evidence-based care across healthcare settings.

## 2. Methodology

This narrative review of the literature aimed to synthesize available evidence and provide practical suggestions and guidance for the clinical management of ascites and pleural effusion in patients with liver cirrhosis and congestive heart failure, with a focus on large-volume paracentesis and coagulation management. Generative AI tool (OpenAI GPT-4.1) was used exclusively for the creation of the graphical abstract but was not used in data processing.

### 2.1. Literature Search

We systematically searched the PubMed (NCBI, NIH. Bethesda, MD, USA), Medline (NIH. Bethesda, MD, USA), Embase (Elsevier, Amsterdam, The Netherlands) and Open Evidence (OpenEvidence Inc., Miami, FL, USA) electronic databases and manually reviewed the relevant society guidelines. The search strategy incorporated both Medical Subject Headings (MeSH) and free-text terms, including: “ascites,” “pleural effusion,” “paracentesis,” “thoracocentesis,” “INR,” “international normalized ratio”, “coagulopathy,” “thrombocytopenia,” “bleeding risk,” and “guidelines”.

Eligible publications were limited to English-language studies indexed between January 2010 and March 2025. The inclusion criteria encompassed randomized controlled trials, observational studies, meta-analyses, and professional society guidelines and expert consensus. The exclusion criteria included studies involving pediatric populations (<18 years), animal studies, case reports, non-peer-reviewed materials, and abstracts only. The quality and relevance of the evidence were qualitatively assessed.

### 2.2. Study Selection and Appraisal

All studies meeting the predefined eligibility criteria were independently evaluated by a multidisciplinary panel of three domain experts in internal medicine, pulmonology, hepatology, and critical care (TS, SD, CB). Titles and abstracts were initially screened to exclude publications that did not directly address the predefined research questions. The full texts of the remaining studies were subsequently retrieved and assessed for final inclusion.

The methodological rigor and reporting transparency of each eligible study were appraised using the Systematic Transparency Assessment in Intervention Reviews (STAIR) checklist, which evaluates the clarity and completeness of key methodological components. Only studies scoring >7.5 points on the STAIR scale were included in the analysis [1].

### 2.3. Results of Literature Screening

**Figure 1:**
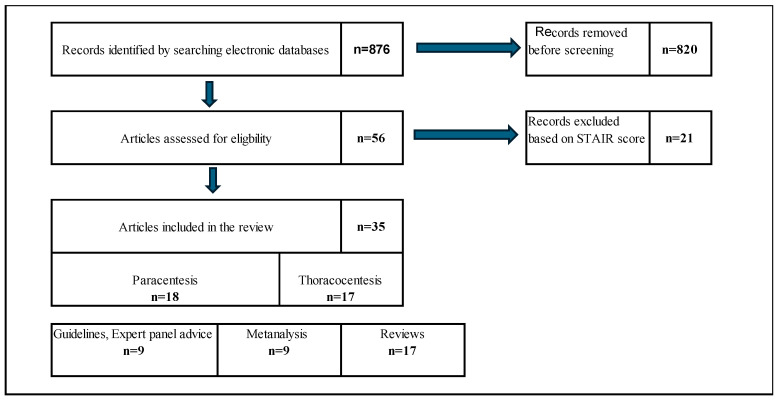
PRISMA flow diagram. PRISMA, Preferred Reporting Items for Systematic Reviews and Meta-Analyses.

The database search identified a total of 876 records. After applying the exclusion criteria, 820 studies were excluded. The remaining 56 articles underwent STAIR quality assessment, and 21 were excluded for scores < 7.5 points. Ultimately, 35 studies met the inclusion criteria: 18 addressed paracentesis, and 17 focused on thoracentesis. Among these, 17 were narrative reviews, nine were meta-analyses, and nine were clinical guidelines or expert consensus recommendations. No randomized controlled trials were identified. The detailed selection process is illustrated in the Preferred Reporting Items for Systematic Reviews and Meta-Analyses (PRISMA) flow diagram (Figure 1).

Findings from the included literature were synthesized into two major domains—abdominal paracentesis for ascites and thoracentesis for pleural effusion—which form the basis of the suggestions presented in the following sections.

## 3. Section I: Abdominal Paracentesis in Patients with Ascites

### 3.1. Indications [1,2,3,4]

Abdominal paracentesis is a key diagnostic and therapeutic procedure for patients with ascites. The indications include:

- Diagnostic assessment: Evaluation of ascitic fluid characteristics, including determination of the serum–ascites albumin gradient (SAAG) to distinguish high (>1.1 g/dL) versus low (<1.1 g/dL) SAAG ascites.
- Evaluation for infection or malignant transformation:
  ○ Rule out spontaneous bacterial peritonitis (SBP).
  ○ Assess for suspected hepatocellular carcinoma–related malignant ascites.
- Symptomatic ascites, such as tense ascites causing respiratory compromise or abdominal discomfort
- Refractory ascites requiring repeated therapeutic drainage
- Adjunctive procedure in patients with ascites contributing to hepatorenal syndrome (performed alongside vasoconstrictor and albumin therapy) [2,5,6].

### 3.2. Clinical Suggestions for Peritoneal Paracentesis

Table 1 lists all recommendations for the pre-procedural preparations, principles of execution of the procedure, and postprocedural care requirements.

### 3.3. Major Complications [3,4,5,6,7,17]

- Hemorrhage (0.3–1%) [3,6]: The incidence of hemorrhage is reduced by 50% with ultrasound (US) guidance [8]. Bleeding during paracentesis typically results from an injury to a branch of the inferior epigastric artery of the abdominal wall. Bleeding can occur in two ways.
  1. Immediate return of blood through the catheter. This usually indicates direct injury to the artery, resulting in immediate blood reflux. This complication generally leads to the development of abdominal wall hematoma [9,10].
  2. Gradual increase in bleeding during the procedure. In such instances, the drainage initially appears serous and progressively becomes hemorrhagic. This suggests arterial bleeding into the peritoneal cavity surrounding the catheter, which leads to increasingly bloody fluid.
- Bowel perforation: This recognized but rare complication occurred in <1% of patients in a large series of diagnostic paracenteses. The risk is higher in patients with intra-abdominal adhesions, prior abdominal surgery, or distorted anatomy due to underlying disease [11,12]. Clinically, bowel perforation may present acutely with the return of fecal material; gas through the paracentesis catheter; or more insidiously with signs of peritonitis, sepsis, or polymicrobial peritoneal infection. Immediate recognition is critical as delayed diagnosis increases peritonitis and sepsis risks. Management depends on the clinical scenario. Stable patients without peritonitis may be managed conservatively with bowel rest and broad-spectrum antibiotics; however, surgical intervention is indicated for patients with generalized peritonitis or clinical deterioration [12,19]. To minimize risk, the needle should be inserted at sites with the lowest likelihood of underlying bowel, such as the left lower quadrant lateral to the rectus abdominis or the midline 2 cm below the umbilicus, and never through surgical scars or areas with suspected adhesions [20].
- SBP during peritoneal paracentesis refers to a peritoneal infection that arises from a direct, identifiable intra-abdominal source that disrupts the integrity of the gastrointestinal or genitourinary tracts. Secondary peritonitis is typically polymicrobial, involving both aerobic and anaerobic organisms, most commonly Gram-negative rods (such as *Escherichia coli* and *Klebsiella* spp.), anaerobes (such as *Bacteroides* spp. and *Clostridium* spp.), and Gram-positive cocci (such as *Enterococcus* spp.). The Infectious Diseases Society of America and the American Society for Microbiology emphasize that, in contrast to SBP, secondary peritonitis is usually polymicrobial and may include anaerobic microbiota, and that peritoneal fluid should be sent for both aerobic and anaerobic cultures in an anaerobic transport system [19]. Diagnosis during paracentesis is suggested by the presence of multiple organisms on Gram staining or culture, elevated ascitic fluid neutrophil count, and biochemical features such as low glucose, high lactate dehydrogenase, and low protein levels. Clinical suspicion should be high in patients with severe abdominal symptoms or evidence of sepsis. Imaging (e.g., computed tomography) is often required to identify the source [20]. Management requires the prompt administration of broad-spectrum antibiotics covering both aerobic and anaerobic organisms and urgent surgical or interventional radiology evaluation to control the infection source, as medical therapy alone is insufficient. Without timely intervention, mortality is high [20,21].
- Post-paracentesis circulatory dysfunction (PICD) manifests as effective hypovolemia and renal impairment, usually after the removal of >5 L. The American College of Radiology states that PICD can develop in up to 80% of patients with cirrhosis if volume expansion (typically using albumin) is not performed at the time of paracentesis. The pathophysiology involves a rapid drop in intra-abdominal pressure, leading to increased venous return, transiently increased cardiac output, and subsequent activation of the renin–angiotensin–aldosterone and sympathetic nervous systems, resulting in decreased effective arterial blood volume and free water retention [22].
- Puncture site leakage following large-volume peritoneal paracentesis is a recognized complication, with reported rates varying from 0% to 23% [12]. The primary cause is persistent ascitic fluid flow through the needle tract, which may be exacerbated by high intra-abdominal pressure, large-volume removal, or technical failure of sealing the skin and subcutaneous tissues after catheter removal. The initial management options include conservative measures [12]. The first-line approach involves the application of a pressure dressing directly over the puncture site, which is effective in most cases. The use of the Z-tract technique during the procedure, in which the skin is displaced before needle insertion to create a zigzag tract, may help prevent leakage, although evidence for its efficacy is limited. If leakage persists, additional options include continued pressure dressings, use of ostomy appliances to collect fluid, or, rarely, placement of a suture at the site [12]. Rarely, persistent or high-volume leaks may require further intervention such as peritoneal drains or surgical closure. Infection should be ruled out if the leak is prolonged or is associated with local erythema.

## 4. Section II: Thoracocentesis in Patients with Pleural Effusion

### 4.1. Indications [8,23,24,25]

- New or unexplained pleural effusion, to differentiate between transudate and exudate based on Light’s criteria
- Suspected infection (empyema or parapneumonic effusion), especially if the effusion is loculated or shows signs of sepsis
- Suspected malignancy, particularly in patients with cancer history or unilateral/bloody effusions
- Suspected tuberculous pleuritis based on the regional epidemiology and clinical presentation
- Symptomatic relief of dyspnea, especially in patients with large pleural effusions causing lung compression and impaired ventilation. This indication is common in malignant effusion, heart failure, or hepatic hydrothorax
- Large effusion with signs of respiratory compromise such as tachypnea, hypoxemia, or use of accessory muscles
- Effusion refractory to medical management; for example: diuretic-resistant pleural effusions in CHF
- Recurrent malignant pleural effusion requiring repeated drainage as a bridge to pleurodesis or indwelling pleural catheter placement

### 4.2. Clinical Suggestions for Thoracocentesis

Table 2 lists all suggestions for the pre-procedural preparations, principles of execution of the procedure, and postprocedural care requirements.

### 4.3. Complications

- Pneumothorax: Pneumothorax, including tension pneumothorax, is the most frequent complication, occurring in approximately 3–6% of cases; However, pneumothorax requiring intervention is rare (<0.5%), especially when ultrasound guidance is used [3,26,27].
- Hemothorax: Bleeding complications, such as chest wall hematoma or hemothorax, are uncommon and are not significantly increased in patients with mild to moderate coagulopathy or thrombocytopenia, particularly when ultrasound guidance is employed [24,28,29,33].
- Re-expansion pulmonary edema: This is a rare but potentially serious complication, with an incidence of <0.1%, and is associated with rapid or large-volume fluid removal, especially if pleural pressures fall below −20 cm H_2_O or if >1.5 L is removed quickly. Symptom-limited drainage is recommended to mitigate this risk [5].
- Secondary infections: Complications following thoracentesis include empyema (pus in the pleural space), parapneumonic effusion (infected pleural fluid associated with pneumonia), and, less commonly, cellulitis or soft tissue infection at the puncture site. These complications are rare when sterile technique is used. Empyema and complicated parapneumonic effusion are clinically significant and often require antimicrobial therapy and drainage. According to the Infectious Diseases Society of America and the American Society for Microbiology, the most common pathogens in pleural space infections are *Streptococcus anginosus*, *Staphylococcus aureus* (including methicillin-resistant *S. aureus* in hospital-acquired cases), anaerobes, and Enterobacterales. Hospital-acquired infections are more likely to involve resistant Gram-negative bacteria [11,12].
- Hemorrhage: Hemorrhage is a recognized but rare complication of pleural effusion paracentesis (thoracentesis) with a risk of significant bleeding (including hemothorax or puncture site bleeding) of approximately ≤1%, as indicated in large meta-analyses and cohort studies. The primary mechanism is misplaced needle or catheter insertion, resulting in laceration of the intercostal artery or its branches, which can lead to chest wall hematoma or hemothorax. Injury to other vascular structures or inadvertent puncture of abdominal organs (e.g., the liver and spleen) is rare but possible, especially for low-lying effusions or with improper techniques. Lack of ultrasound guidance, poor knowledge of the local anatomy, and multiple needle passes increase the risk. Vascular ultrasonography with color Doppler can help avoid vessel injury [26,28].

## 5. Thoracocentesis and Paracentesis in the Coagulopathic Patient

Thoracentesis and paracentesis are among the most common invasive bedside procedures performed in internal medicine, hepatology, interventional radiology, and critical care. Both procedures are consistently categorized as low bleeding risk by major professional societies, including the SIR, hepatology societies such as the American Association for the Study of Liver Diseases (AASLD), and pleural procedure experts [13,30,31,34,35].

Historically, abnormal standard coagulation tests (thrombocytopenia, prolonged INR), or ongoing antiplatelet/anticoagulant therapy, triggered automatic correction before performing these procedures, usually with platelet transfusion, fresh frozen plasma (FFP), or interruption of antithrombotic drugs. However, this practice is increasingly challenged. Contemporary data from large observational series and consensus guidelines show that clinically significant procedure-related bleeding is rare (<1%), even in patients with thrombocytopenia, elevated INR from cirrhosis, or therapeutic antithrombotic use [13,14,15,16,17,18,30,32,34,35]. At the same time, preemptive correction (platelets, FFP) carries real risks of transfusion-associated circulatory overload, transfusion-related acute lung injury, allergic reactions, exposure to donor products, and procedural delay, as well as cost [13,14,34,35]. Current expert guidance, therefore, favors a physiology-based, ultrasound-guided, restrictive transfusion strategy rather than a lab-number–driven, prophylactic correction strategy [13,14,15,16,30,32,34,35]. Five questions should be addressed:

### 5.1. Severe Thrombocytopenia: Does It Need to Be Corrected, and at What Platelet Threshold?

Early dogma required platelet levels to be >50,000/µL before performing “any invasive procedure.” More recent data specific to paracentesis and thoracentesis do not support that universal threshold. In a prospective/observational thoracentesis cohort of 312 patients, 42% had an abnormal coagulation profile (thrombocytopenia, elevated INR, or antithrombotic therapy). No patient developed hemothorax attributable to the procedure, and no patient experienced clinically significant post-procedural hemoglobin drop associated with low platelet count [32]. Similar findings were reproduced in other pleural procedure safety series, where thrombocytopenia did not predict major bleeding when thoracentesis was ultrasound-guided and performed by experienced operators [15,16,32].

For paracentesis, large retrospective and prospective series in patients with cirrhosis, a group frequently experiencing thrombocytopenia, showed procedure-related major bleeding rates < 1%, even with platelet counts well below 50,000/µL [13,14,15,18,30,34,35]. Importantly, in pooled analyses summarized in the SIR consensus, most bleeding complications actually occurred in patients whose platelet counts were above 50,000/µL, suggesting that the absolute platelet number alone is not a reliable predictor of clinically significant hemorrhage for these specific low-risk procedures [13,15,30].

A strong biologic rationale supports this concept. Cirrhosis produces “rebalanced hemostasis”, in which reductions in procoagulant factors are offset by decreases in endogenous anticoagulants and increases in factor VIII and von Willebrand factor. As a result, global hemostasis may remain functionally compensated despite abnormal conventional labs and thrombocytopenia [14,34,35]. Thus, low platelets in cirrhosis do not translate linearly into uncontrolled procedural bleeding.

These pathophysiologic and clinical data underpin the platelet thresholds adopted in contemporary society guidelines.

The SIR 2019 consensus guidelines classify thoracentesis and paracentesis as low bleeding risk and state that these procedures can generally be performed without prophylactic platelet transfusion when the platelet count is ≥20,000/µL [13,30]. Platelet transfusion is mainly suggested for platelet counts < 20,000/µL or in patients showing clinical evidence of an active systemic bleeding tendency (e.g., disseminated intravascular coagulation).

AASLD and hepatology guidance for ascites management in cirrhosis advises that routine correction of thrombocytopenia is not required before diagnostic or therapeutic paracentesis. These documents often frame > 50,000/µL as reassuring but explicitly note that evidence does not support systematic platelet transfusion at any fixed threshold for low-risk procedures [34,35].

Critical care/American College of Chest Physician (CHEST) guidance on transfusion in the ICU suggests a restrictive platelet transfusion strategy and recommends against routine prophylactic platelet transfusion before low bleeding risk bedside procedures (explicitly including paracentesis and thoracentesis) in thrombocytopenic but non-bleeding critically ill adults [14,18].

Practical interpretation (synthesized thresholds):

Platelets ≥ 50,000/µL: universally considered safe to proceed without prophylactic platelet transfusion for ultrasound-guided thoracentesis or paracentesis [13,14,15,16,18,32,34,35].

Platelets 20,000–50,000/µL: SIR and multiple observational series support proceeding without prophylactic platelet transfusion, provided there is no active bleeding, no disseminated coagulopathy, and a small-caliber, ultrasound-guided technique is used [13,15,16,30,32].

Platelets < 20,000/µL (especially <10–20 K/µL) or clinical evidence of mucosal/spontaneous bleeding: Many experts consider transfusing platelets or delaying an elective procedure. This recommendation is based on expert consensus, physiologic reasoning, and observational experience, not randomized trial data [13,14,15,16,30].

### 5.2. Prolonged INR: How Should It Be Managed, and When (If Ever) Should FFP Be Administered?

INR was designed to monitor vitamin K antagonist therapy, not to quantify global hemostasis in advanced liver disease. In cirrhosis, increased INR reflects decreased hepatic synthesis of procoagulant factors but does not account for concomitant decreases in natural anticoagulants and increases in prohemostatic mediators such as factor VIII and von Willebrand factor [14,34,35]. Thus, elevated INR in cirrhosis systematically overestimates the procedural bleeding risk.

Retrospective and prospective studies of paracentesis and thoracentesis in cirrhotic or medically coagulopathic patients have demonstrated no meaningful association between INR values up to ~2.5–3.0 and procedure-related clinically significant bleeding [13,15,16,18,30,32]. Pooled analyses report major bleeding rates of approximately 0.2% for paracentesis and 0.5% for thoracentesis in patients with “abnormal coagulation”, generally defined as INR > 1.5 and/or platelets < 50 × 10^9^/L [13,15,16,30].

Given the poor predictive value of INR in cirrhosis, empirically transfusing FFP to “correct” INR before paracentesis or thoracentesis does not reduce bleeding risk. Moreover, FFP transfusion carries meaningful risks, including volume overload (critical in tense ascites and pleural effusion), transfusion reactions, transfusion-related acute lung injury (TRALI), and procedural delay [13,14,34,35].

For these reasons, AASLD guidance on ascites management and paracentesis explicitly recommends against routine prophylactic FFP transfusion solely to normalize INR in patients with cirrhosis undergoing paracentesis [34,35]. Interventional radiology (SIR) consensus likewise discourages routine FFP administration for mildly to moderately elevated INR when performing low-risk procedures such as thoracentesis and paracentesis [13,30]. Critical care/CHEST transfusion guidance supports a restrictive plasma strategy and advises against prophylactic FFP before low-risk bedside procedures in non-bleeding critically ill adults [14].

FFP (or prothrombin complex concentrate) may be considered in select situations, including: marked pharmacologic coagulopathy, such as clearly supratherapeutic warfarin effect (e.g., INR > 3.5–4.0), when partial reversal is required before an urgent large-volume procedure; overt disseminated intravascular coagulation (DIC) with evidence of clotting factor consumption; or active major bleeding.

These represent targeted, individualized correction, not blanket INR normalization [13,14,34,35].

Practical interpretation:

- INR ≤ ~2.0 in a hemodynamically stable cirrhotic or medically coagulopathic patient without active bleeding: proceed with ultrasound-guided paracentesis or thoracentesis without prophylactic FFP [13,14,15,16,30,32,34,35].
- INR > 3.5 due to warfarin over-anticoagulation, or frank DIC with active bleeding: consider etiology-based reversal (vitamin K, PCC/FFP, cryoprecipitate if hypofibrinogenemia) on a case-by-case basis [13,14,30,34,35].

### 5.3. Patients on Anticoagulants (Warfarin, DOACs) and Antiplatelet Agents (Aspirin, Clopidogrel, Ticagrelor): Should Therapy Be Stopped Pre-Procedure?

**Warfarin.** Traditional perioperative teaching mandated holding warfarin until the INR fell < 1.5 before performing any invasive procedure. This practice has been challenged for thoracentesis and paracentesis. In observational thoracentesis cohorts, patients on warfarin, often with therapeutic INR in the 2.0–3.0 range, did not experience increased rates of clinically significant bleeding, and no hemothorax was directly attributable to the procedure when ultrasound guidance was used [15,16,32]. Based on these data, the SIR consensus classifies thoracentesis and paracentesis as low bleeding-risk and does not require correction of a therapeutic warfarin INR (nor routine FFP transfusion) before these procedures [13,30].

Practical approach:

- Urgent procedures: (e.g., large symptomatic effusion, diagnostic paracentesis for suspected spontaneous bacterial peritonitis), most experts proceed without reversing therapeutic warfarin (INR in target range ~2–3.5).
- Markedly supratherapeutic INR (>3.5) due to warfarin overdose: many clinicians hold warfarin and/or administer vitamin K; in urgent cases with perceived high bleeding risk, PCC or FFP may be considered. These strategies are based on expert consensus rather than RCT-level evidence [13,14,15,16,30].
- Elective procedures: INR ~2 should be the limit for pre-procedural correction. Routine “bridging” with heparin is not indicated for these short interruptions during low-risk procedures.

**DOACs (direct oral anticoagulants: apixaban, rivaroxaban, edoxaban, dabigatran.**

No randomized trials focused specifically on DOAC continuation vs. interruption for thoracentesis/paracentesis have been performed. Thus, evidence comes from retrospective safety series and extrapolation from broader periprocedural DOAC management frameworks. SIR and pleural procedure expert statements consider thoracentesis and paracentesis to be low bleeding-risk and acknowledge that these procedures are frequently performed urgently without DOAC reversal, with very low rates of clinically significant bleeding [13,14,18,30].

Practical approach:

- Urgent/indicated thoracentesis or paracentesis: most clinicians proceed without delaying solely for DOAC washout [1,2,15,16,17,18].
- Elective procedures in clinically stable patients with normal renal function: many clinicians hold one DOAC dose (~24 h) beforehand as a precaution. This reflects expert practice rather than evidence from thoracentesis-/paracentesis-specific trials [13,15,16,17,18,30].

Heparin bridging is not recommended for these brief interruptions.

**Antiplatelet agents (aspirin, clopidogrel, ticagrelor)**

Classic perioperative rules advise stopping clopidogrel/ticagrelor for 5–7 days before nearly any invasive procedure. However, evidence specific to thoracentesis and paracentesis does not support this level of caution for these low-risk bedside procedures.

In a prospective thoracentesis cohort, patients on antiplatelet therapy (including aspirin and clopidogrel) showed no excess major bleeding, and no hemothorax events were directly attributed to thoracentesis [15,16,32]. Additional retrospective pleural procedure data and expert consensus statements from interventional radiology and pleural medicine similarly report that continuation of aspirin, and in many cases even P2Y12 inhibitors, does not meaningfully increase clinically significant bleeding when thoracentesis is performed under ultrasound guidance with small-caliber catheters [13,15,16,18,30].

Importantly, stopping P2Y12 inhibitors (clopidogrel/ticagrelor) in patients with recent coronary stenting can precipitate stent thrombosis, which carries a far higher mortality risk than the extremely low rate of major bleeding associated with thoracentesis or paracentesis [15,16].

Practical approach:

- Aspirin monotherapy: generally continue; no need to stop before thoracentesis or paracentesis.
- Clopidogrel/ticagrelor:
  - Urgent, clinically indicated procedure: most experts proceed without interruption [13,15,16,30].
  - Elective procedures in low cardiac-risk patients: some practitioners prefer to defer or briefly hold P2Y12 inhibitors for 72 h; this remains a conservative institutional habit rather than an evidence-based requirement.
- Dual antiplatelet therapy after recent stent: should not be interrupted without cardiology input, as thrombosis risk greatly outweighs procedural bleeding risk [15,16,18].

### 5.4. Which Patients Are Actually “High Bleeding Risk” for These Procedures?

Although thoracentesis and paracentesis are inherently low bleeding-risk procedures, certain clinical states increase danger. Guidance documents and expert consensus highlight the following:

1. **Global consumptive or systemic coagulopathy**
  - DIC
  - Severe hyperfibrinolysis
  - Hypofibrinogenemia (<100–120 mg/dL)
  These states represent uncontrolled, active bleeding biology, not just an abnormal INR in compensated cirrhosis. In such patients, targeted correction (e.g., cryoprecipitate for low fibrinogen, platelets for profound thrombocytopenia) before even “low-risk” procedures is reasonable [13,14,34,35].
2. **Profound thrombocytopenia (<20,000/µL) with evidence of clinical bleeding.**
  Below ~10–20 K/µL, spontaneous mucosal and intracranial bleeding risk is intrinsically high, independent of procedural trauma. This population is considered high risk; many experts transfuse platelets or delay non-urgent procedures [13,14,15,16,17,30].
3. **More invasive pleural or abdominal access than a standard tap.**
  Data supporting safety apply to ultrasound-guided thoracentesis and paracentesis using a small-bore catheter. When larger-bore chest tubes, tunneled drains, thoracoscopy, or closed pleural biopsy are planned, several consensus statements consider these “moderate” or “high bleeding-risk” procedures and recommend more conservative coagulation targets such as platelets > 50,000/µL or correction of major coagulopathy [13,15,16,17,18,30].
4. **Non–ultrasound-guided (“blind”) access, distorted anatomy, or operator inexperience**.
  Multiple passes, loculated effusions, adhesions, or blind attempts increase the chance of vascular injury. Ultrasound guidance is repeatedly emphasized as the standard of care because it reduces vascular puncture and bleeding [13,15,16,30,32].
5. **Ongoing thrombolytic therapy or very recent systemic fibrinolysis**.
  Patients actively receiving thrombolytics are generally considered to be at high risk for procedure-related bleeding, even for nominally low-risk procedures [14,18].

### 5.5. Where Do Guidelines and Expert Statements Disagree?

Despite broad convergence toward a restrictive, physiology-based approach, important inconsistencies remain:

**(a)**  **Platelet threshold**
  - **SIR 2019 consensus (interventional radiology):**
    Thoracentesis and paracentesis are low risk; platelet transfusion is not routinely required unless platelets < 20,000/µL or there is active bleeding [13,30].
  - **Hepatology guidelines (AASLD; British Society of Gastroenterology/BASL):**
    Strongly discourage routine correction, but the language is more conservative, often framing >50,000/µL as reassuring and implying that transfusion “may be considered” below that level, despite acknowledging limited evidence [34,35].
  - **Critical care/CHEST transfusion guidance:**
    Endorses a restrictive transfusion strategy and does not impose a universal numeric platelet cutoff above which taps are “safe,” instead focusing on clinical bleeding phenotype and procedure risk category [14]. In practice, hepatology and internal medicine ward culture often remains more cautious below 50 K, while interventional radiology and ICU practice are comfortable down to ~20 K in stable patients.
**(b)**  **INR correction with FFP**
  - AASLD and hepatology guidance:
    Elevated INR in cirrhosis does not predict procedural bleeding and should not be routinely corrected with FFP before paracentesis [34,35].
  - SIR consensus:
    Similarly discourages routine pre-procedural FFP for mildly or moderately elevated INR in low-risk procedures [13,30].
  - Legacy perioperative/surgical policies:
    Many hospitals still enforce “INR < 1.5” rules before any invasive procedure and require prophylactic FFP in patients with cirrhosis. This practice persists despite a lack of supportive evidence and is increasingly viewed as outdated for these specific bedside procedures [13,14,15,16,30].
**(c)**  **Management of antiplatelet and anticoagulant therapy**
  - Interventional radiology/pleural expert statements:
    Thoracentesis and paracentesis are low risk; aspirin, clopidogrel, ticagrelor, and even therapeutic warfarin or DOACs often do not need to be stopped in urgent situations [13,15,16,18,30,31].
  - Traditional perioperative anticoagulation algorithms:
    Still commonly instruct clinicians to stop clopidogrel/ticagrelor for 5–7 days before almost any invasive procedure and to hold DOACs pre-procedure. These algorithms are largely extrapolated from surgery, neuraxial anesthesia, or moderate/high-risk procedures, not from ultrasound-guided fluid aspiration [8,9,10]. Thus, cardiology (concerned about stent thrombosis), hepatology (concerned about delaying life-saving paracentesis for spontaneous bacterial peritonitis), and interventional radiology (comfortable with low needle-track bleeding risk) tend to accept continued antithrombotic therapy, while surgical/anesthesia checklists are sometimes more conservative.
**(d)**  **Viscoelastic testing: thromboelastography/rotational thromboelastometry (TEG/ROTEM)**

Some hepatology and critical-care groups advocate TEG or ROTEM to evaluate “true” hemostatic capacity in cirrhosis and guide selective transfusion, instead of relying on INR or platelet count alone [14,15,34]. This approach can reduce unnecessary blood product use. However, routine access to TEG/ROTEM is not universal, and not all guidelines have embedded viscoelastic-guided decision-making for thoracentesis/paracentesis, producing variability between tertiary liver centers and community hospitals [14,15,34].

**Figure 2:**
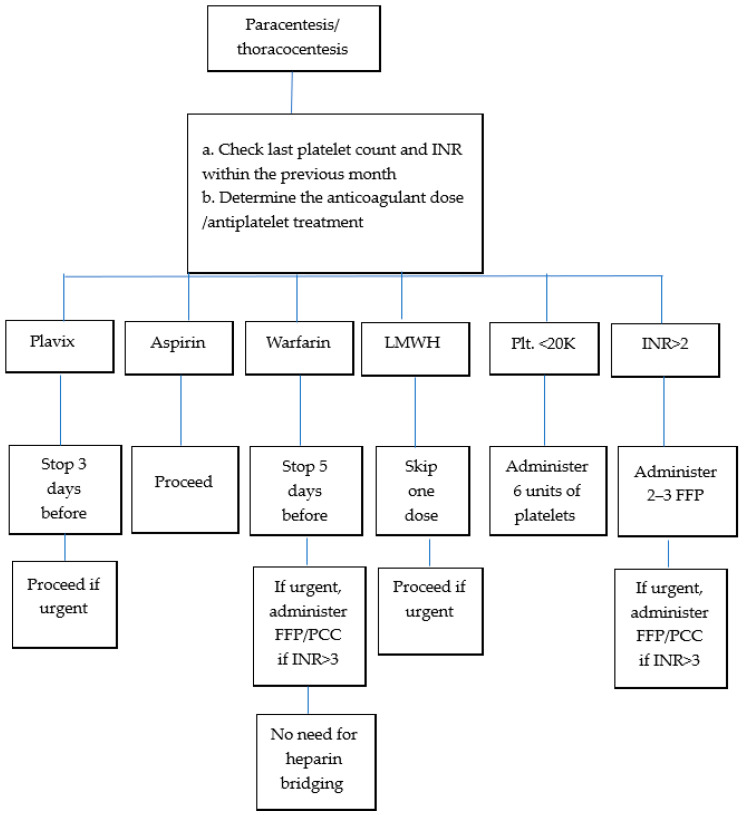
Algorithm for pre-procedure management of coagulopathy. Plt, platelets; INR, international normalized ratio; FFP, fresh frozen plasma; PCC, prothrombin complex concentrate; LMWH, low-molecular-weight heparin.

Figure 2 presents an algorithm summarizing the suggestions required for coagulopathy management before performing paracentesis or thoracocentesis.

## 6. Discussion

Thoracentesis and paracentesis are low bleeding-risk procedures, even in the presence of moderate thrombocytopenia, elevated INR from cirrhosis, and ongoing antiplatelet or anticoagulant therapy. The use of POCUS has further reduced procedural complications. Modern evidence and expert consensus from hepatology, interventional radiology, and critical care demonstrate that major hemorrhagic complications are uncommon (<1%).

Platelet count alone is a poor predictor of bleeding, and safe practice extends down to platelet counts in the 20,000–50,000/µL range without transfusion in stable, non-bleeding patients. Increased INR in cirrhosis does not reflect true procedural bleeding risk, and prophylactic FFP is generally unnecessary for patients with INR ≤ ~2 and no evidence of active bleeding. Likewise, aspirin, P2Y12 inhibitors, therapeutic warfarin, and DOACs do not universally require interruption for urgent thoracentesis or paracentesis because the procedural bleeding risk is extremely low, while the thrombotic risk associated with stopping therapy may be high. The novelty of this work lies in several key components: The first is the presentation of combined pleural and peritoneal puncture guidance side by side in one document. Although complications may differ between the two anatomical spaces, the systematic approach to pre-procedural and intraprocedural management is nearly identical. Second, our work provides a concise and practical algorithm for managing drainage procedures in the setting of coagulopathy—an issue that concerns physicians from multiple disciplines prior to performing these interventions. Third, the structured, evidence-based approach to managing puncture procedures and coagulopathy facilitates a shift in these procedures from inpatient hospital settings to community-based facilities and day-care units, thereby enhancing accessibility and resource efficiency. The literature review and the proposed protocol represent an important step toward standardizing practice for clinicians across specialties who perform these procedures. The implementation of the proposed guide is also applicable to remote regions and resource-limited settings. Ultimately, the requirements for a safe and successful paracentesis are minimal: a large-bore peripheral intravenous catheter, a smartphone-compatible ultrasound transducer—technology that is already widely available worldwide and access to a basic laboratory capable of performing platelet count and INR testing, reserved for exceptional or high-risk cases only.

Despite these strengths, the narrative review format introduces inherent limitations. Much of the available evidence is based on retrospective studies, case series, and single-country experiences. Several of the studies are subject to publication bias which likely contributed to the lack of strong consensus among existing guidelines. The lack of prospective outcome validation for the proposed protocol emphasizes the need for prospective registries, randomized multicenter controlled trials (RCTs) and high-quality meta-analyses to confirm these suggestions and strengthen future guideline development.

## 7. Conclusions

Although both abdominal and pleural LVP are considered low-risk interventions, meticulous attention to coagulation status, imaging guidance, and postprocedural monitoring is essential for reducing complications. The guidelines proposed here align with the current literature and have been adapted to high-risk populations. The emphasis on detailed documentation ensures accountability and safety. Adherence to these suggestions can reduce procedural complications, optimize patient outcomes, especially in complex cases involving coagulopathies or anticoagulant therapy, and direct standardization of safety care.

## Figures and Tables

**Table 1:** **Clinical guidance** for paracentesis [3,4,5,6,7,8,9,10,11,12,13,14,15,16,17,18].

Section	Suggestion
**Preprocedural suggestions**	
1. Informed consent	Obtain verbal consent and document. Written consent should be obtained only if required by institutional policy.
2. First-time paracentesis	Provide a full explanation of the risks in the patient’s native language. Document the explanation, clinician’s name, and language used.
3. Exclude acute infection	Postpone the procedure if active infection is suspected.
4. Coagulation guidance	No repeat tests if no coagulopathy history and normal test results in the past month. a. DOACs: Stop 24 h prior (skip one dose). Proceed if emergency drainage required. b. Warfarin: Stop 5 d prior; confirm INR < 2.0. If emergency, correct with FFP/PCC above INR-3.0. c. Clexane (LMWH): Hold 12 h (skip last dose). Proceed if emergency drainage required. d. Aspirin: proceed. e. Plavix: Stop 72 h prior. Proceed if emergency drainage required. f. Platelet count < 20,000/μL: Administer 6 units. g. INR > 2.0: Administer ≤ 3 FFP units. (~0.3 INR correction per unit) [1]. If emergency correct with FFP/PCC above INR-3.0. h. TEG: Optional if available.
5. Baseline vitals	Record temperature, pulse, BP, and oxygen saturation.
**Technique suggestions**	
1. Ultrasound guidance	Mandatory for all procedures.
2. Needle placement	Avoid the midline. Insert 8 cm lateral to the midline and 5 cm above the pubic symphysis or 2 cm below the umbilicus.
3. Drainage system	Use either an 8F Seldinger kit or 18G cannula.
4. Volume	No restriction on the amount of peritoneal fluid drained (unlike pleural effusion).
5. Diagnostic fluid sampling	Always analyze fluid for cell count. If neutrophil count is >250/mm^3^ → suspect SBP. If a catheter is in place → perform culture testing.
6. Device options	Either an 8F Seldinger set or 18G peripheral catheter is acceptable.
7. Initial/diagnostic studies	Analyze fluid for: cell count, culture, albumin, TP, amylase, BNP (optional), cytology.
8. Albumin administration	Administer 8–10 g albumin for each 1 L of drainage (only >5 L) (to prevent paracentesis-induced circulatory dysfunction)
**Post-paracentesis care**	
1. Observation	Monitor patients for 1 h after catheter removal
2. Bed rest	Keep patient in bed for the first 30 min post removal.
3. Discharge criteria	Discharge only after documentation of stable BP and HR. Ensure parameters are recorded.
4. Leakage from site.	If leakage occurs, place a pressure bandage. If leakage does not stop, a single suture may be placed. Remove suture after 1 week.
5. Resuming anticoagulation/anti-platelets	Resume anticoagulants (including clopidogrel) the day after the procedure.
**Bleeding management**	
1. Bloody aspirate	Serosanguinous initial fluid is acceptable. If bloody fluid follows clear drainage → stop immediately. If the initial aspirate is blood → abort and remove the catheter.
2. Bleeding response	a. Monitor for 4 h with vitals assessed every hour. b. Repeat hemoglobin testing after 1 h. Perform blood type testing.
**Admission criteria post-paracentesis**	
1. SBP	Confirmed SBP diagnosis.
2. Hemodynamic instability	Hemodynamic changes or a drop in hemoglobin level > 1 g/dL.
3. Severe pain or hematoma	VAS ≥ 7 + expanding hematoma → consider urgent abdominal CT angiography.
**Documentation checklist**	
1. Indication	Document the indication for the procedure
2. Consent	Obtain and document verbal or written consent.
3. Physical/POCUS findings	Record the physical exam and POCUS findings.
4. Technique	Document the insertion technique and exact place.
5. Vital signs	Record pre- and post-procedure vital signs.
6. Anticoagulation	Document anticoagulation status and last dose timing.
7. Laboratory test results	Include relevant laboratory test results and hemostasis assessment

Abbreviations: BNP, brain natriuretic peptide; BP, blood pressure; Clexane, brand name for low molecular weight heparin (LMWH); CT, computed tomography; DOACs, direct oral anticoagulants; FFP, fresh frozen plasma; INR, international normalized ratio; LMWH, low molecular weight heparin; SBP, spontaneous bacterial peritonitis; TP, total protein; VAS, visual analog scale; POCUS, point-of-care ultrasound.

**Table 2:** Clinical guidance for pleural drainage [13,16,23,25,26,27,28,29,30,31,32].

Section	Recommendation
**Preprocedural requirements and suggestions**	
1. Imaging	Localize effusion using POCUS.
2. Access point	Two intercostal spaces below the fluid peak in the posterior mid-clavicular line with the patient seated, arms forward. Above the rib.
3. Needle/drainage Kit	20–21G cannula or 6–8F Seldinger catheter.
4. Coagulation guidance	No repeat tests if no coagulopathy history and normal test results in the past month. a. DOACs: Stop 24 h prior. (skip one dose). Proceed if emergency drainage required. b. Warfarin: Stop 5 d prior; confirm INR < 2.0. If emergency drainage required, correct with FFP/PCC only if INR > 3.0. c. Clexane (LMWH): Hold 24 h (2 doses). Proceed if emergency drainage required. d. Aspirin: Continue [13]. e. Plavix: Stop 72 h prior [13,27]. Proceed if emergency drainage required. f. Platelet count < 20,000/μL: Administer 6 units. g. In elective procedure if INR > 2.0 administer ≤ 3 FFP units. If emergency drainage required, correct with FFP/PCC only if INR > 3.0. h. TEG: Optional if available.
**Procedure guidance**	
1. Bilateral drainage	Do not perform on the same day.
2. Volume limit	Do not exceed 1.5 L drainage per session [25].
3. Drainage method	Use gravity drainage. Avoid vacuum suction [2].
4. Anesthesia	Use 1% lidocaine for local anesthesia [25].
5. Diagnostic testing	Send pleural fluid for pH, LDH, glucose, protein, cytology, and BNP assessments.
**Post-procedure care**	
1. Vital signs	Monitor hourly for 2 h.
2. Imaging	Chest radiography (AP + LAT) 1 h post-procedure [25].
3. Anticoagulation/anti-platelets	Restart the next day.
**Admission criteria post procedure**	
New dyspnea/hypoxemia	Pneumothorax in X-ray. blood aspiration or aspirated fluid became bloody during drainage-suspected hemothorax.
Hemodynamic instability	Suspect Tension pneumothorax (emergency thoracocentesis)
**Documentation checklist**	
1. Indication	Document the indication for the procedure.
2. Consent	Obtain and document verbal or written consent.
3. Physical/POCUS Findings	Record the physical exam and POCUS findings.
4. Technique	Document the insertion technique.
5. Vital Signs	Record pre- and post-procedure vital signs.
6. Anticoagulation	Document anticoagulation status and last dose timing.
7. Laboratory test results	Include relevant laboratory test results and hemostasis assessment.

Abbreviations: AP: anteroposterior; BNP: B-type natriuretic peptide; Clexane: brand name for enoxaparin, a low-molecular-weight heparin; DOACs: direct oral anticoagulants; FFP: fresh frozen plasma; PCC-prothrombin complex concentrate; INR: international normalized ratio; LAT: lateral; LDH: lactate dehydrogenase; LMWH: low-molecular-weight heparin; Plavix: brand name for clopidogrel; POCUS: point-of-care ultrasound; TEG: thromboelastography.

## Data Availability

Not applicable.

## Abbreviations

The following abbreviations are used in this manuscript:

AASLD	American Association for the Study of Liver Diseases
AP	anteroposterior
BASL	British Association for the Study of the Liver
BNP	B-type natriuretic peptide
BP	blood pressure
CHEST	American College of Chest Physicians
CHF	congestive heart failure
CT	computed tomography
DIC	disseminated intravascular coagulation
DOAC	direct oral anticoagulant
DOACs	direct oral anticoagulants
FFP	fresh frozen plasma
HR	heart rate
HRS	hepatorenal syndrome
ICU	intensive care unit
INR	international normalized ratio
LAT	lateral
LDH	lactate dehydrogenase
LMWH	low-molecular-weight heparin
LVP	large-volume paracentesis
MeSH	Medical Subject Headings
PCC	prothrombin complex concentrate
PICD	postparacentesis circulatory dysfunction
POCUS	point-of-care ultrasound
PRISMA	Preferred Reporting Items for Systematic Reviews and Meta-Analyses
RCTs	randomized controlled trials
ROTEM	rotational thromboelastometry
SAAG	serum–ascites albumin gradient
SBP	spontaneous bacterial peritonitis
SIR	Society of Interventional Radiology
TEG	thromboelastography
TP	total protein
US	ultrasound
VAS	visual analog scale
